# Iron deficiency and the effectiveness of the BNT162b2 vaccine for SARS-CoV-2 infection: A retrospective, longitudinal analysis of real-world data

**DOI:** 10.1371/journal.pone.0285606

**Published:** 2023-05-22

**Authors:** Lilac Tene, Avraham Karasik, Gabriel Chodick, Dora I. A. Pereira, Henrik Schou, Sandra Waechter, Udo-Michael Göhring, Hal Drakesmith

**Affiliations:** 1 Maccabi Institute for Research & Innovation, Maccabi Healthcare Services, Tel Aviv, Israel; 2 CSL Vifor, Glattbrugg, Switzerland; 3 Medical Research Council Human Immunology Unit, MRC Weatherall Institute of Molecular Medicine, University of Oxford, Oxford, United Kingdom; Children’s National Hospital, George Washington University, UNITED STATES

## Abstract

**Background:**

Iron plays a key role in human immune responses; however, the influence of iron deficiency on the coronavirus disease 2019 (COVID-19) vaccine effectiveness is unclear.

**Aim:**

To assess the effectiveness of the BNT162b2 messenger RNA COVID-19 vaccine in preventing severe acute respiratory syndrome coronavirus 2 (SARS-CoV-2) infection and COVID-19–related hospitalization and death in individuals with or without iron deficiency.

**Methods:**

This large retrospective, longitudinal cohort study analyzed real-world data from the Maccabi Healthcare Services database (covering 25% of Israeli residents). Eligible adults (aged ≥16 years) received a first BNT162b2 vaccine dose between December 19, 2020, and February 28, 2021, followed by a second dose as per approved vaccine label. Individuals were excluded if they had SARS-CoV-2 infection before vaccination, had hemoglobinopathy, received a cancer diagnosis since January 2020, had been treated with immunosuppressants, or were pregnant at the time of vaccination. Vaccine effectiveness was assessed in terms of incidence rates of SARS-CoV-2 infection confirmed by real-time polymerase chain reaction assay, relative risks of COVID-19–related hospitalization, and mortality in individuals with iron deficiency (ferritin <30 ng/mL or transferrin saturation <20%). The two-dose protection period was Days 7 to 28 after the second vaccination.

**Results:**

Data from 184,171 individuals with (mean [standard deviation; SD] age 46.2 [19.6] years; 81.2% female) versus 1,072,019 without (mean [SD] age 46.9 [18.0] years; 46.2% female) known iron deficiency were analyzed. Vaccine effectiveness in the two-dose protection period was 91.9% (95% confidence interval [CI] 83.7–96.0%) and 92.1% (95% CI 84.2–96.1%) for those with versus without iron deficiency (*P* = 0.96). Of patients with versus without iron deficiency, hospitalizations occurred in 28 and 19 per 100,000 during the reference period (Days 1–7 after the first dose), and in 19 and 7 per 100,000 during the two-dose protection period, respectively. Mortality rates were comparable between study groups: 2.2 per 100,000 (4/181,012) in the population with iron deficiency and 1.8 per 100,000 (19/1,055,298) in those without known iron deficiency.

**Conclusions:**

Results suggest that the BNT162b2 COVID-19 vaccine is >90% effective in preventing SARS-CoV-2 infection in the 3 weeks after the second vaccination, irrespective of iron-deficiency status. These findings support the use of the vaccine in populations with iron deficiency.

## Introduction

The BNT162b2 messenger RNA (mRNA) coronavirus disease 2019 (COVID-19) vaccine demonstrated 95% efficacy in preventing wild-type severe acute respiratory syndrome coronavirus 2 (SARS-CoV-2) infection, with a two-dose regimen, in a phase 3 placebo-controlled trial [1]. Real-world observational studies have validated these findings, with two-dose effectiveness rates of 86% to 95% in preventing SARS-CoV-2 infection [2–6] and of 87% to 92% in preventing COVID-19–related hospitalization or severe disease [2, 5]. Further real-world data are needed to ascertain whether effectiveness is high in individuals with underlying conditions that may predispose to COVID-19–related hospitalization and death.

Iron plays a key role in innate and adaptive immunity and is necessary for immune cell growth, proliferation, and differentiation [7–9]. Several studies suggest that iron deficiency leads to impaired immune responses [8–12]. Cohort studies show that iron deficiency also affects vaccine effectiveness. For example, elderly patients with iron deficiency have a poorer immune response to influenza vaccination than those without [13]. Anemia and iron deficiency decrease the immune response of infants to the combined diphtheria, tetanus, and whooping cough vaccine, the pneumococcal vaccine, and the measles vaccine [10, 14, 15], whereas iron supplementation augments the immune response to measles vaccination [14].

Iron is required for growth of almost all human pathogens. It is therefore plausible that iron therapy and/or iron deficiency influence infection risk. Studies provide moderate-quality evidence that intravenous iron might increase risk of infection [16, 17], and labeling for injectable iron supplements indicates that they must be used with caution in cases of acute or chronic infection. Recently, iron depletion has been proposed as an adjuvant therapy for COVID-19 infection [18, 19]. However, evidence establishing an association between iron and infection is inconclusive [16]. Initial reports from small retrospective studies suggest that patients with iron deficiency may be at increased risk of severe COVID-19 disease. In a retrospective analysis of individuals hospitalized with COVID-19, those with moderate or severe anemia or iron-deficiency anemia (IDA) were at increased risk of death compared with those with normal hemoglobin levels [20, 21]. Another retrospective study suggested that low serum iron levels were associated with COVID-19 severity [22], while a study involving patients undergoing hemodialysis found that higher ferritin levels were associated with an improved response to SARS-CoV-2 vaccination [23]. However, due to the lack of randomized controlled trial data, causality cannot be inferred, and it is plausible that low serum iron is a marker of disease severity due to underlying inflammation associated with COVID-19.

The European Hematology Association advises clinicians to correct iron deficiency before COVID-19 vaccination [24]. However, more data are needed on the effectiveness of COVID-19 vaccines in individuals with iron deficiency to validate this guidance [25].

In Israel, a high vaccination coverage of >70% of adults permitted analysis of vaccine effectiveness in specific populations considered to be at higher risk from COVID-19. The primary objective of our study, which used real-world data from the Israeli Maccabi Healthcare Services (MHS) database, was to investigate the effectiveness of the BNT162b2 mRNA COVID-19 vaccine in preventing SARS-CoV-2 infection and COVID-19–related hospitalization and death in individuals with known iron deficiency compared with those without known iron deficiency. Exploratory objectives included assessment of vaccine effectiveness according to age, sex, and comorbidities.

## Materials and methods

### Study design

This retrospective, longitudinal cohort study used patient-level data from the MHS database, a state-mandated health maintenance organization covering 2.6 million members or 25% of residents in Israel. This database includes fully anonymized data on demographics, anthropometrics, community clinic and hospital diagnoses, medication dispensing, and central laboratory measurements. A risk-interval design was used [26], which differs from traditional methods as incidence rates for risk and non-risk time periods are compared only among vaccinated individuals. As only vaccinated individuals are included, biases introduced by comparing vaccinated and unvaccinated populations are minimized. Risk estimates are also unaffected by any potential population imbalances as the same patient cohort is followed over time. The study was conducted in accordance with the Good Clinical Practice Guidelines of the International Conference on Harmonisation of Technical Requirements for Registration of Pharmaceuticals for Human Use, and with Declaration of Helsinki ethical principles for medical research involving human subjects. The study protocol was approved by the Maccabi Healthcare Services Institutional Review Board (IRB) on June 27, 2021 (0056-21-MHS). The need for informed consent was waived by the IRB for this retrospective database-based study.

### Study population

Data were extracted for members >16 years of age who were registered in the MHS database before March 1, 2020, and who received a first dose of the BNT162b2 vaccine between December 19, 2020, and February 28, 2021. Iron deficiency was determined using the most recent blood test results from up to 2 years before vaccination to a maximum of 3 months after vaccination (except during pregnancy). Among patients with test results available, known iron deficiency was declared if ferritin was <30 ng/mL, or transferrin saturation (TSAT) <20%. A ferritin level <30 ng/mL with or without a TSAT <20% is presumed to reflect pure or true iron deficiency, as a low TSAT can be found in both ID and inflammation, but with an accompanying low serum ferritin level presumably reflects ID. Iron deficiency was further stratified as absolute iron deficiency (ferritin <15 ng/mL) or functional iron deficiency (TSAT <20%). IDA was defined as mild to moderate if hemoglobin levels were ≥8 g/dL and <12 g/dL (females) or ≥8 g/dL and <13 g/dL (males), and as severe if hemoglobin levels were <8 g/dL.

Clinical characteristics and comorbidities were recorded around the time of vaccination, including body mass index, history of cancer, and chronic diseases (specifically diabetes, cardiovascular disease [CVD], chronic kidney disease [CKD; level 3b and above], and hypertension). Individuals were excluded from the study if they had SARS-CoV-2 infection before vaccination. In order to increase the specificity of our ID criteria, we also excluded patients with a documented hemoglobinopathy, had received a cancer diagnosis since January 2020, had been treated with immunosuppressants, or were pregnant at the time of vaccination.

### Outcomes

Outcomes included a positive SARS-CoV-2 test result obtained via real-time–polymerase chain reaction (RT-PCR), COVID-19–related hospitalization, and COVID-related mortality rate. Primary care physicians determined the presence of symptomatic infection through the routine follow-up of symptoms via a questionnaire after registration of a positive test result. COVID-19 hospitalization was defined hospital admission within 1 month of a positive test result, or admission at any date to a designated coronavirus ward after registration of a positive result. Deaths were considered to be COVID-19 related if they occurred within 3 months of a positive RT-PCR test result.

### Data analysis

Data were analyzed during three study periods: the reference period (Days 1–7 after the first dose, assuming no vaccine protection in that period) [27], the one-dose protection period (Days 13–21 after the first vaccine dose, and no later than Day 27 in case of a delayed second vaccine dose), and the two-dose protection period (Days 7–28 after the second vaccine dose). Vaccine effectiveness was evaluated by comparing incidence rates of RT-PCR–confirmed SARS-CoV-2 infection between these periods via Kaplan-Meier analysis and generalized linear models. A negative binomial distribution was applied, using an offset of log-link and log-daily number of individuals at risk, to scale SARS-CoV-2 infection counts to the daily population at risk. Censoring included death, leaving the MHS database, or end of follow-up. Analyses were stratified by age, sex, and comorbidity for the main analysis, and were compared between individuals with and without known iron deficiency (i.e., those with ferritin ≥30 ng/mL or TSAT ≥20%). Sensitivity analyses were performed for iron deficiency subtypes and iron-supplementation use (ie, with or without receiving supplementation at least once in the 2 years before or up to 3 months after vaccination). Differences in mortality and hospitalization were calculated using relative risk with 95% confidence intervals (CIs), adjusted for period, iron-deficiency status, age, sex, and comorbidities. Analyses were conducted using IBM-SPSS, version 27 (Armonk, NY, USA), and R packages magrittr, readtext, dplyr, ggplot2, tidyverse, survival, forestplot, and survminer.

## Results

Overall, 1,256,190 individuals vaccinated with the BNT162b2 vaccine were included (S1 Fig), comprising 184,171 with iron deficiency (mean [standard deviation; SD] age 46.2 [19.6] years) and 1,072,019 without known iron deficiency (mean [SD] age 46.9 [18.0] years) (Table 1). Among individuals with versus without known iron deficiency, there were higher proportions of women (81.2% [149,614/184,171] versus 46.2% [495,505/1,072,019]), fewer individuals who smoked (13.7% [25,150/184,171] versus 19.1% [204,745/1,072,019]), and higher frequencies of comorbidities. Baseline characteristics were generally similar between individuals with iron deficiency, absolute iron deficiency, functional iron deficiency, and mild to moderate IDA, with some differences observed in the smaller population of individuals with severe IDA. Iron-related parameters and comorbidities for individuals with iron deficiency are shown in S1 Table, by iron deficiency type and iron-supplement use. Among individuals with iron deficiency, those taking iron supplementation had lower mean ferritin and TSAT levels, and higher frequencies of comorbidity, than those not taking iron supplements.

**Table 1:** Baseline characteristics.

Characteristic	No Known ID (n = 1,072,019)	ID^a^ (n = 184,171)	Absolute ID (n = 37,253)	Functional ID (n = 64,278)	Mild IDA (n = 41,080)	Severe IDA (n = 334)
**Sex, n (%)**						
Male	576,514 (53.8)	34,557 (18.8)	3594 (9.6)	18,330 (28.5)	8237 (20.1)	85 (25.4)
Female	495,505 (46.2)	149,614 (81.2)	33,659 (90.4)	45,948 (71.5)	32,843 (79.9)	249 (74.6)
**Age, mean (SD), y**	46.90 (17.95)	46.19 (19.61)	42.30 (17.70)	52.25 (19.44)	51.60 (21.24)	58.85 (20.84)
**Smoking status, n (%)**						
Yes	204,745 (19.1)	25,150 (13.7)	4918 (13.2)	9230 (14.4)	4702 (11.4%)	55 (16.5)
Quit smoking	73,836 (6.9)	13,435 (7.3)	2286 (6.1)	6131 (9.5)	3401 (8.3%)	28 (8.4)
**Socioeconomic status,**^**b**^ **median (IQR)**	7 (5–8)	7 (5–8)	6 (5–8)	7 (5–8)	6 (5–8)	6 (5–8)
**Iron supplementation, n (%)**	60,744 (5.7)	51,690 (28.1)	15,568 (41.8)	21,620 (33.6)	17,266 (42.0%)	235 (70.4)
Tablets	55,526 (5.2)	38,970 (21.2)	10,328 (27.7)	14,840 (23.1)	11,858 (28.9)	67 (20.1)
Injections	3256 (0.3)	7765 (4.2)	3262 (8.8)	4112 (6.4)	3182 (7.7)	105 (31.4)
Tablets and injections	1962 (0.2)	4955 (2.7)	1978 (5.3)	2668 (4.2)	2226 (5.4)	63 (18.9)
**Comorbidities, n (%)**						
Obesity	217,175 (21.5)	40,732 (22.7)	7808 (21.5)	17,674 (28.0)	10,539 (26.2)	87 (26.3)
Diabetes mellitus	91,405 (8.5)	26,054 (14.1)	4019 (10.8)	13,110 (20.4)	8943 (21.8)	84 (25.1)
**Cardiovascular disease**	57,703 (5.4)	14,627 (7.9)	1704 (4.6)	7880 (12.3)	5360 (13.0)	48 (14.4)
**Chronic kidney disease (level ≥3b)**	12,503 (1.2)	5061 (2.7)	358 (1.0)	3558 (5.5)	3173 (7.7)	57 (17.1)
**Hypertension**	216,894 (20.2)	42,744 (23.2)	6496 (17.4)	20,751 (32.3)	13,542 (33.0)	124 (37.1)
**Charlson Comorbidity Index, median (IQR)**	0 (0–1)	0 (0–1)	0 (0–1)	0 (0–2)	0 (0–3)	(0–5)

ID = iron deficiency; IDA = iron-deficiency anemia; IQR = interquartile range; SD = standard deviation.

^a^ Not all patients with ID had absolute or functional ID as they may have had ferritin levels of 15–30 ng/mL. Similarly, not all patients with ID had anemia, as they may have had hemoglobin levels ≥12 g/dL in males or ≥13 g/dL in females. SI conversion factors: To convert ferritin to μg/L, multiply value by 1.0; to convert hemoglobin to g/L, multiply value by 10.0.

^b^ Derived for commercial purposes by Points Location Intelligence using geographic information systems and data, such as expenditures related to retail chains, credit cards, and housing. This score highly correlates with socioeconomic status as measured by the Central Bureau of Statistics and is ranked as follows: 1 (lowest) to 10 (highest) socioeconomic status.

**Fig 1:**
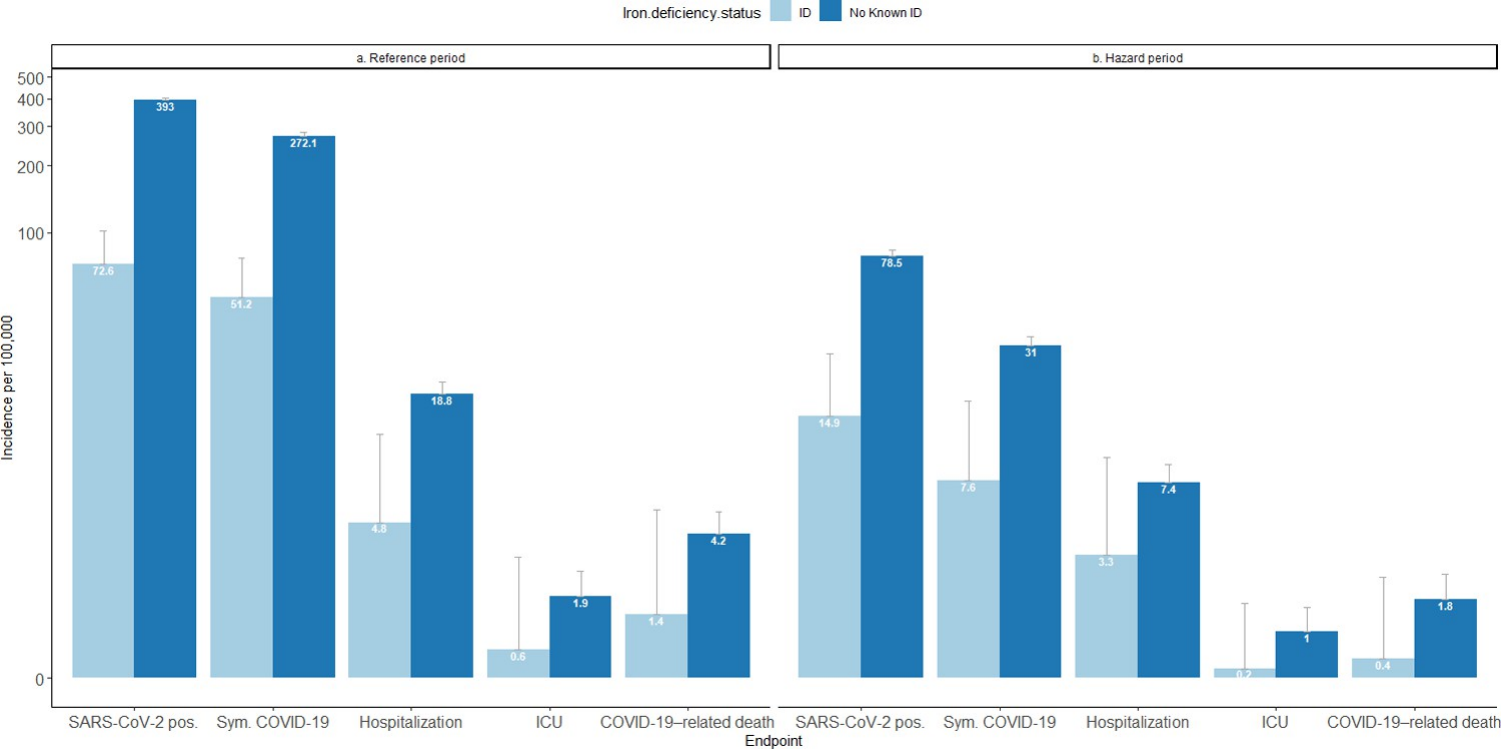
COVID-19 outcomes, according to study period. COVID-19 = coronavirus disease 2019; ICU = intensive care unit; ID = iron deficiency.

**Fig 2:**
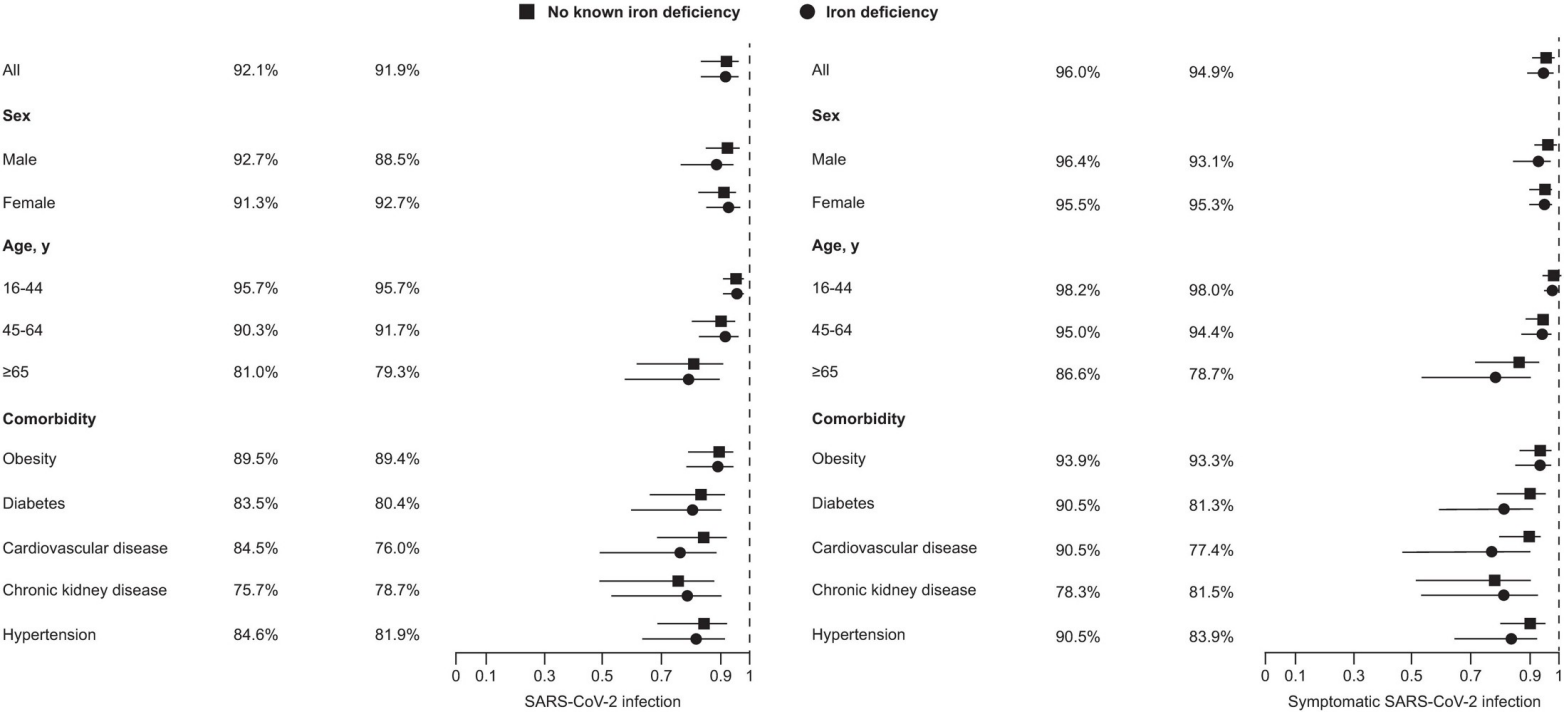
Two-dose vaccine effectiveness against severe acute respiratory syndrome coronavirus 2 (SARS-CoV-2 infection) (left) and symptomatic SAS-CoV-2 infection (right).

The incidence of SARS-CoV-2 infection in individuals with or without known iron deficiency reduced from 422 per 100,000 (778/184,171) and 392 per 100,000 (4213/1,072,019) during the reference period, to 88 per 100,000 (160/181,012) and 80 per 100,000 (841/1,055,298) during the two-dose protection period, respectively (Fig 1). One-dose vaccine effectiveness against SARS-CoV-2 infection was numerically lower but not statistically different for individuals with ID, versus those without known iron deficiency: 39.1% (95% CI –17.3% to 68.4%) versus 44.9% (95% CI –5.9% to 71.4%), respectively (*P*≈1). Two-dose vaccine effectiveness for ID versus without known ID was 91.9% (95% CI 83.7–96.0%) and 92.1% (95% CI 84.2–96.1%; *P* = 0.96) (Fig 2).

A similar pattern of vaccine effectiveness was seen against SARS-CoV-2 infection among individuals with or without known iron deficiency, irrespective of sex, obesity, and hypertension (Fig 1). Vaccine effectiveness in various patient subgroups by age, in individuals with and without known iron deficiency, is shown in S2 Fig.

SARS-CoV-2 infection rates for each iron deficiency subtype are shown in Table 2. Two-dose vaccine effectiveness was >90% among individuals with absolute or functional ID, and among those with mild or severe IDA (S2 Table). Vaccine effectiveness across ID subtypes according to age, sex, and comorbidities is shown in S2 Table. Within the population with ID, there was no significant difference in vaccine effectiveness against SARS-CoV-2 infection for individuals taking iron supplements (90.7%; 95% CI 80.2–95.6%), versus those with no record of iron supplementation (93.8%; 95% CI 86.9–97.1%; *P* = 0.34) (S3 Table). Among those receiving injectable iron, vaccine effectiveness was 87.2% (95% CI 72.1–94.1%). The absence of statistical difference in two-dose vaccine effectiveness between iron-supplementation subgroups was observed for both sexes and all age groups.

**Table 2:** COVID-19 characteristics of vaccine recipients with ID, according to ID type and supplement use.

	ID (n = 184,171)	Absolute ID (n = 37,253)	Functional ID (n = 64,278)	Mild IDA (n = 41,080)	ID Without Supplements (n = 132,481)	ID With Supplements (n = 51,690)
**COVID-19 infection**						
Reference period	778 (422)	182 (489)	275 (428)	207 (504)	555 (419)	223 (431)
Two-dose protection period	160 (87)	25 (67)	77 (120)	54 (131)	101 (76)	59 (114)
**COVID-19 hospitalization**						
Reference period	51 (28)	5 (13)	29 (45)	30 (73)	24 (18)	27 (52)
Two-dose protection period	35 (19)	5 (13)	25 (39)	19 (46)	19 (14)	16 (31)
**COVID-19 ICU admission**						
Reference period	6 (3.3)	1 (2.7)	3 (4.7)	5 (12)	1 (0.8)	5 (9.7)
Two-dose protection period	2 (1.1)	0	2 (3.1)	1 (2.0)	0	2 (3.9)
**COVID-19–related death**						
Reference period	15 (8.1)	1 (2.7)	8 (12)	10 (24)	5 (3.8)	10 (19)
Two-dose protection period	4 (2.2)	1 (2.7)	2 (3.1)	3 (7.3)	1 (0.8)	3 (5.8)

Data shown as n (n per 100,000).

COVID-19 = coronavirus disease 2019; ICU = intensive care unit; ID = iron deficiency; IDA = iron-deficiency anemia.

Two-dose vaccine effectiveness against symptomatic COVID-19 infection was similar for individuals with (94.9%; 95% CI 89.2–97.6%) and without iron deficiency (96.0%; 95% CI 91.5–98.1%; *P* = 0.69) (Fig 2).

COVID-19–related hospitalization decreased between the reference and two-dose protection periods in populations with and without iron deficiency, indicating a protective effect of vaccination (Fig 1). During the reference period, hospitalizations occurred in 28 per 100,000 (51/184,171) of individuals with iron deficiency and 19 per 100,000 (202/1,072,019) of those without known iron deficiency. During the two-dose protection period, hospitalizations occurred in 19 per 100,000 (35/181,012) of individuals with iron deficiency and 7.5 per 100,000 (79/1,055,298) of those without iron deficiency. COVID-19–related intensive care unit (ICU) admission also decreased between the reference and protection periods for both groups, although ICU admission was rare overall (Fig 1). COVID-19 hospitalization rates for each iron-deficiency subtype are shown in Table 2.

In the multivariate analyses, vaccination was a significant protective factor against COVID-19–related hospitalization (adjusted odds ratio [AOR] 0.60; 95% CI 0.369–0.574) (Table 3). Other significant risk factors for hospitalization included older age, male sex, presence of CKD, and hypertension. In this model, iron deficiency was a significant risk factor for COVID-19–related hospitalization (AOR 1.555; 95% CI 1.209–1.999). When vaccinated individuals with ID were stratified according to iron-supplement use, iron-deficiency status was a significant independent risk factor for hospitalization only for those taking supplements (AOR 2.037; 95% CI 1.456–2.851) (Table 3). Additional stratification by iron-supplement type showed that iron deficiency only remained a risk factor for individuals receiving injectable supplements (AOR 3.186; 95% CI 2.041–4.972), and was not a significant risk factor in those taking oral supplements (AOR 1.405; 95% CI 0.885–2.230).

**Table 3:** Univariate and multivariate logistic regression models for hospitalization in the overall population.

	Univariate Model, AOR (95% CI)	Multivariate Models, AOR (95% CI)^a^	
**Protection period** ^**b**^	0.458 (0.367–0.571)	0.60 (0.369–0.574)	0.460 (0.369–0.574)
**ID** ^**c**^	1.783 (1.400–2.270)	1.555 (1.209–1.999)	-
**ID without supplements** ^**c**^	1.239 (0.899–1.708)	-	1.272 (0.918–1.762)
**ID with supplements** ^**c**^	3.181 (2.307–4.384)	-	2.037 (1.456–2.851)
**Age**	1.076 (1.069–1.083)	1.130 (1.106–1.154)	1.130 (1.106–1.154)
**Sex** ^**d**^	0.817 (0.665–1.003)	0.771 (0.622–0.955)	0.775 (0.625–0.959)
**Diabetes mellitus** ^**e**^	3.836 (3.057–4.814)	1.282 (1.001–1.642)	1.272 (0.993–1.629)
**Cardiovascular disease** ^**e**^	4.062 (3.143–5.249)	0.964 (0.726–1.278)	0.956 (0.720–1.268)
**Chronic kidney disease** ^**e**^	14.699 (11.204–19.284)	3.462 (2.550–4.701)	3.360 (2.471–4.570)
**Hypertension** ^**e**^	3.132 (2.550–3.847)	9.744 (3.065–30.976)	9.850 (3.104–31.255)
**Hypertension × age**	-	0.961 (0.946–0.977)	0.961 (0.946–0.976)

AOR = adjusted odds ratio; CI = confidence interval; ID = iron deficiency.

^**a**^Multivariate models are mutually adjusted for all listed variables in the relevant column.

^b^Compared with the reference period.

^c^Compared with “without known ID”.

^d^Compared with males.

^e^Compared with those without disease.

Twenty-three deaths occurred during the protection period, limiting data analysis for this outcome. The proportion of COVID-19–related deaths decreased numerically between the reference and two-dose protection periods among individuals with and without iron deficiency (Fig 1). During the reference period, COVID-19–related deaths occurred in 8.1 per 100,000 (15/184,171) of individuals with iron deficiency and 4.2 per 100,000 (45/1,072,019) of those without known iron deficiency. During the two-dose protection period, COVID-19 mortality rates were 2.2 per 100,000 (4/181,012) among those with iron deficiency and 1.8 per 100,000 (19/1,055,298) among those without known iron deficiency.

## Discussion

In this large, retrospective analysis, the BNT162b2 mRNA COVID-19 vaccine had a high level of effectiveness in the 3 weeks following second vaccination in patients with documented iron deficiency, supporting its use in protecting such patients from SARS-CoV-2 infection and COVID-19–related hospitalization and death. To our knowledge, this is the first large study to use real-world data to determine the impact of iron deficiency on a COVID-19 vaccine in the general population. Overall, we found no difference in the effectiveness of two doses of this vaccine in preventing SARS-CoV-2 infection in individuals with and without known iron deficiency. The two-dose vaccine effectiveness rate of >90% observed in both populations is in line with the two-dose vaccine protection seen during a phase 3 vaccine efficacy trial (95%) and in real-world vaccine-effectiveness studies (92–94%) [1–3, 6].

While our study showed that vaccination was a significant protective factor against COVID-19 related hospitalization, risk factors for hospitalizations included older age, male sex, presence of CKD, hypertension and iron deficiency. Within the population with known iron deficiency, receipt of injectable iron supplements was associated with a three-fold increased risk of hospitalization, compared to patients with IS, whereas, ID patients on oral iron supplements did not show such significantly higher risk. One explanation for the increased hospitalization rate in patients with iron deficiency receiving injectable iron supplements is exacerbating the infection. Iron is required for viral replication and can be acquired by modifying host-cell iron homeostasis [16, 19]. Indeed, the labeling for injectable iron supplements indicates that parenteral iron must be used with caution in cases of active or chronic infection, and treatment decisions should therefore account for the risks and benefits of iron supplementation in this patient group [16]. In addition, the increased risk of hospitalization among patients on injectable iron supplements is a potential channeling bias. These patients were more likely to have underlying co-morbid conditions that increased their risk of hospitalization compared to patients on oral iron supplements. Although the multivariate analyses were adjusted for diabetes, CVD, CKD, and hypertension, there may have been confounders that were unadjusted for in the model, which could have affected these findings. Moreover, individuals taking iron supplementation had lower ferritin and TSAT levels, indicating more severe iron deficiency. Further studies are warranted to evaluate the association between iron supplementation and severity of COVID-19 infection.

Although there were no age-related differences in BNT162b2 mRNA COVID-19 vaccine efficacy noted during the phase 3 clinical trial [1], we observed a lower two-dose vaccine effectiveness for SARS-CoV-2 infection and symptomatic infection among individuals aged ≥65 years than in younger vaccine recipients, with similar findings in those with and without iron deficiency. This age-related difference is in line with results of a real-world study involving the general population [3]. Among individuals with diabetes, those with iron deficiency showed more exaggerated age-related variation in vaccine effectiveness than those without known iron deficiency, with the highest effectiveness observed in those aged ≤44 years. Similar observations were made regarding patients with iron deficiency who had CVD or CKD. However, the small number of individuals limits interpretation of these data due to lack of statistical power.

Our study has several limitations. This retrospective analysis of real-world data was, by its nature, not randomized. Therefore, attributing causality remains a major limitation due to the non-random assignment of subjects to treatment. It is particularly problematic in our database analyses where the clinical context of prescribing injectable rather than oral iron supplements is not fully understood. We compared a population with iron deficiency with the general population, rather than a matched cohort without iron deficiency, however the analysis was stratified by age, sex, and several important comorbidities. Misclassification is another study limitation. Individuals with undiagnosed iron deficiency at the time of vaccination could have been included in the population without known iron deficiency, since group allocation was performed without a recent iron parameter test. Iron deficiency was ascertained using blood test results from up to 2 years before and up to 3 months after vaccination. Overall, 58% of the tests were from 6 months before or 3 months after vaccination; 44% were from 3 months before or after vaccination. As this time period is relatively long, some individuals may have had iron deficiency for a considerable time before vaccination, and subsequently had this corrected by iron supplementation or by treatment of underlying health conditions, and therefore may have had normalized iron levels at the time of vaccination. As there is a lack of consensus guidelines for iron deficiency diagnosis [28], we defined functional iron deficiency based on TSAT levels, although some guidelines recommend using a combination of parameters [29]. In addition, it is not known when patients were taking their iron supplements and, in several cases, it may have been some time before they were vaccinated against or contracted SARS-CoV-2. Absolute iron deficiency was defined as ferritin <15 ng/mL, in line World Health Organization guidelines [30]. Notably, we evaluated short-term vaccine effectiveness (lasting 3 weeks after the second vaccine dose) but not long-term protection. A possible effect of iron deficiency on duration of protection is important because protection from two-dose COVID-19 vaccination programs may wane in the following months [31]. Further studies are required to evaluate vaccine safety among patients with iron deficiency and to investigate preliminary safety signals reported by others [32]. Our findings can be extrapolated to mRNA COVID-19 vaccines only; further investigations are needed to assess the effectiveness of other vaccine types in iron-deficient populations. Despite these limitations, the cohort size should provide reassurance regarding the validity of our primary findings. Finally, mortality in this study, which was conducted from December 2020 to February 2021, was very low (<10 per 100,000), which precluded statistical analysis of vaccine effectiveness by iron status.

## Conclusions

In this retrospective, longitudinal analysis of registry data, the BNT162b2 COVID-19 vaccine was >90% effective in preventing SARS-CoV-2 infection and also reduced COVID-19–related hospitalization in a real-world setting, in individuals with and without known iron deficiency, in the 3 weeks after their second vaccination. Findings support the use of this vaccine in iron-deficient populations.

## Supporting information

S1 Fig Data extraction participant flowchart. (PDF)

S2 Fig Two-dose vaccine effectiveness against severe acute respiratory syndrome coronavirus 2 (SARS-CoV-2) infection, according to age group. (PDF)

S1 Table ID components and comorbidities of vaccine recipients with ID, according to ID type and supplement use. (DOCX)

S2 Table Two-dose vaccine effectiveness against SARS-CoV-2 infection, according to ID type. (DOCX)

S3 Table Two-dose vaccine effectiveness in people with ID, according to iron supplementation. (DOCX)
