# Supplementary figures and images for: Iron deficiency and the effectiveness of the BNT162b2 vaccine for SARS-CoV-2 infection: A retrospective, longitudinal analysis of real-world data
Source: PLoS One. 2023 May 22;18(5):e0285606. doi: 10.1371/journal.pone.0285606 (PMC10202294; doi:10.1371/journal.pone.0285606)

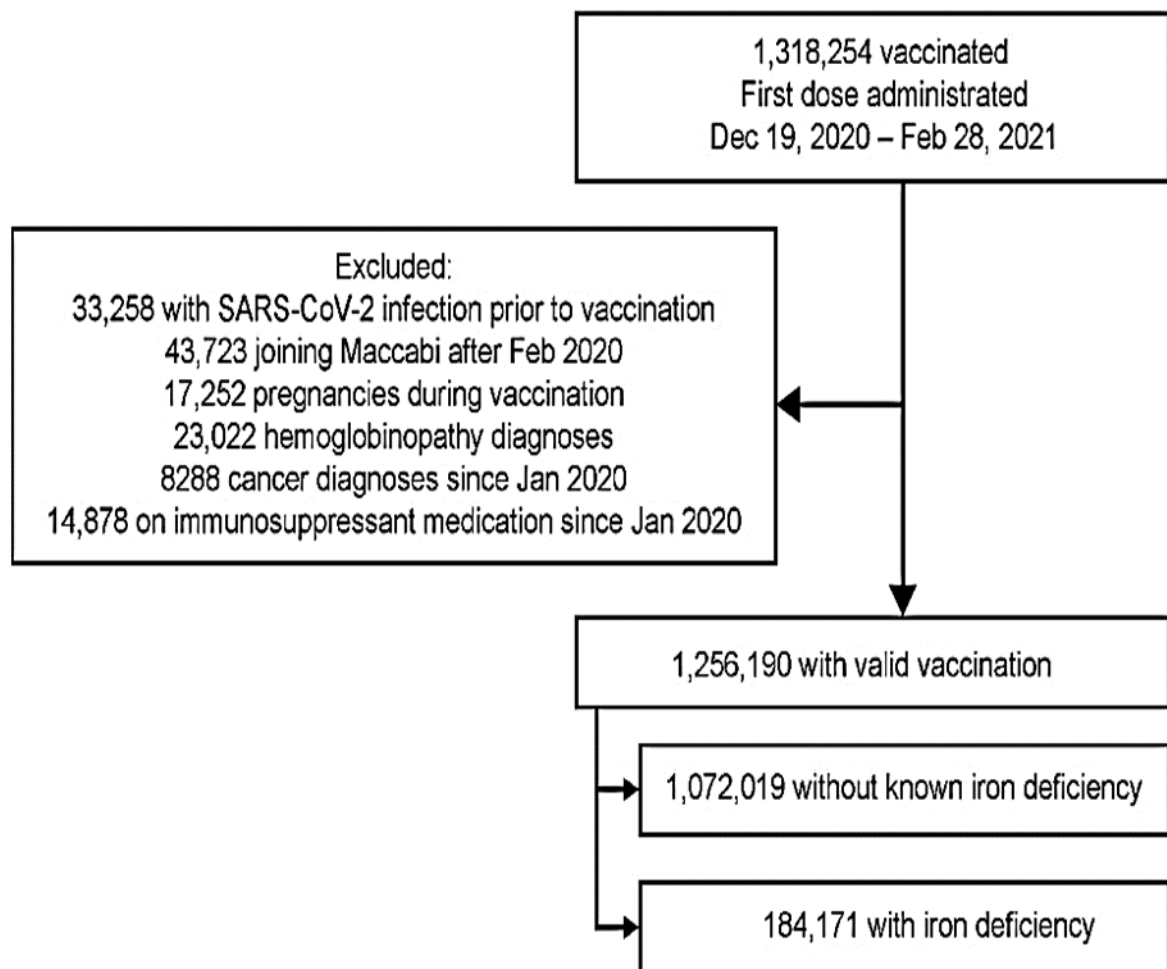

Supplement: S1 Fig — (PDF) [file pone.0285606.s001.pdf]

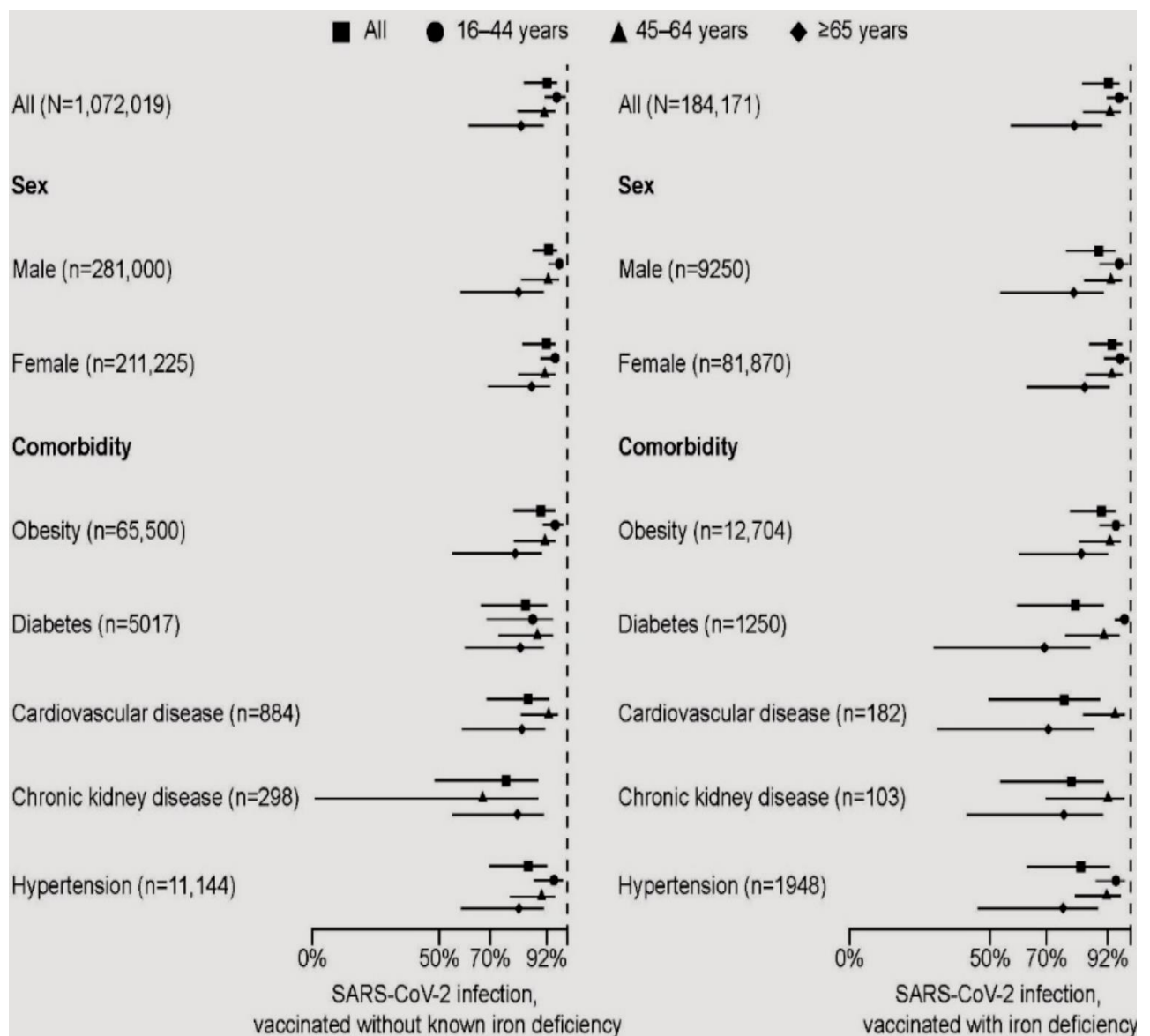

Supplement: S2 Fig — (PDF) [file pone.0285606.s002.pdf]
