# Supplementary material for: Iron deficiency and the effectiveness of the BNT162b2 vaccine for SARS-CoV-2 infection: A retrospective, longitudinal analysis of real-world data
Source: PLoS One. 2023 May 22;18(5):e0285606. doi: 10.1371/journal.pone.0285606 (PMC10202294; doi:10.1371/journal.pone.0285606)
Supplement: S1 Table — (DOCX) [file pone.0285606.s003.docx]

| **ID (n=184,171)** | **Absolute ID (n=37,253)** | **Functional ID**  **(n=64,278)** | | **Mild IDA**  **(n=41,080)** | **ID Without Supplements (n=132,481)** | **ID With Supplements (n=51,690)** |
| --- | --- | --- | --- | --- | --- | --- |
| **Ferritin^a^, mean (SD),** ng/mL | 19.58 (6.14) (n=148,773) | 11.32 (1.94) (n=37,253) | 18.63 (6.26) (n=28,880) | 16.90 (6.35)  (n=33,560) | 20.30 (5.91) (n=105,827) | 17.78 (6.32) (n=42,946) |
| **TSAT, mean (SD), %** | 13.99 (4.11)  (n=64,278) | 10.38 (4.49) (n=8939) | 13.99 (4.11) (n=64,278) | 12.52 (4.58)  (n=16,122) | 14.72 (3.69) (n=42,658) | 12.55 (4.48) (n=21,620) |
| **Hemoglobin^a^, mean (SD),** g/dL | 12.91 (1.33) (n=183,973) | 12.26 (1.34) (n=37,209) | 12.95 (1.48) (n=64,241) | 11.27 (0.85)  (n=41,080) | 13.06 (1.26) (n=132,313) | 12.53(1.43) (n=51,660) |
| **Comorbidities, n (%)** | | | | | | |
| Obesity | 40,732 (22.7) | 7808 (21.5) | 17,674 (28.0) | 10,539 (26.2) | 26,944 (20.3) | 13,788 (26.7) |
| Diabetes mellitus | 26,054 (14.1) | 4019 (10.8) | 13,110 (20.4) | 8943 (21.8) | 15,884 (12.0) | 10,170 (19.7) |
| Cardiovascular disease | 14,627 (7.9) | 1704 (4.6) | 7880 (12.3) | 5360 (13.0) | 8613 (6.5) | 6014 (11.6) |
| Chronic kidney disease (level ≥3b) | 5061 (2.7) | 358 (1.0) | 3558 (5.5) | 3173 (7.7) | 2306 (1.7) | 2755 (5.3) |
| Hypertension | 42,744 (23.2) | 6496 (17.4) | 20,751 (32.3) | 13,542 (33.0) | 27,435 (20.7) | 15,309 |

ID = iron deficiency; IDA = iron-deficiency anemia; SD = standard deviation; TSAT = transferrin saturation.

**a** SI conversion factor: To convert ferritin to μg/L, multiply value by 1.0; to convert hemoglobin to g/L, multiply value by 10.0.
