# Supplementary material for: Iron deficiency and the effectiveness of the BNT162b2 vaccine for SARS-CoV-2 infection: A retrospective, longitudinal analysis of real-world data
Source: PLoS One. 2023 May 22;18(5):e0285606. doi: 10.1371/journal.pone.0285606 (PMC10202294; doi:10.1371/journal.pone.0285606)
Supplement: S2 Table — (DOCX) [file pone.0285606.s004.docx]

|  | **No Known ID (n=1,072,019)** | **ID (n=184,171)** | **Absolute ID (n=37,253)** | **Functional ID (n=64,278)** | **Mild IDA (n=41,080)** | **Severe IDA (n=334)** |
| --- | --- | --- | --- | --- | --- | --- |
| **All** | 92.1 (84.2–96.1) | 91.9 (83.7–96.0) | 95.1 (89.6–97.7) | 90.1 (79.1–95.3) | 90.7 (80.2–95.6) | 92.1 (84.2–96.1) |
| **Age**, y | | | | | | |
| 16–44 | 95.7 (91.3–97.8) | 95.7 (91.4–97.9) | 96.1 (91.7–98.2) | 96.1 (91.6–98.2) | 94.7 (88.5–97.5) | 95.7 (91.3–97.8) |
| 45–64 | 90.3 (80.6–95.2) | 91.7 (83.2–95.9) | 94.6 (88.2–97.5) | 91.5 (81.8–96.1) | 94.1 (87.1–97.3) | 90.3 (80.6–95.2) |
| ≥65 | 81.0 (61.7–90.5) | 79.3 (57.7–89.9) | 89.6 (73.9–95.8) | 75.8 (46.8–89.0) | 79.1 (53.8–90.5) | 81.0 (61.7–90.5) |
| **Sex** | | | | | | |
| Male | 92.7 (85.5–96.4) | 88.5 (76.7–94.4) | 91.9 (81.0–96.6) | 87.9 (73.8–94.4) | 80.9 (56.9–91.5) | 92.7 (85.5–96.4) |
| Female | 91.3 (82.5–95.6) | 92.7 (85.4–96.4) | 95.5 (90.5–97.9) | 91.2 (81.2–95.8) | 92.7 (84.4–96.6) | 91.3 (82.5–95.6) |
| **Obesity** | 89.5 (79.0–94.8) | 89.4 (78.5–94.8) | 90.9 (79.6–95.9) | 90.7 (79.9–95.7) | 91.2 (80.8–96.0) | 89.5 (79.0–94.8) |
| **Diabetes mellitus** | 83.5 (66.8–91.8) | 80.4 (59.9–90.5) | 88.7 (73.4–95.2) | 80.5 (57.2–91.1) | 79.6 (54.0–90.9) | 83.5 (66.8–91.8) |
| **Hypertension** | 84.6 (69.0–92.3) | 81.9 (63.2–91.1) | 92.3 (82.6–96.6) | 80.6 (57.9–91.1) | 81.4 (59.1–91.5) | 84.6 (69.0–92.3) |
