# Supplementary material for: Iron deficiency and the effectiveness of the BNT162b2 vaccine for SARS-CoV-2 infection: A retrospective, longitudinal analysis of real-world data
Source: PLoS One. 2023 May 22;18(5):e0285606. doi: 10.1371/journal.pone.0285606 (PMC10202294; doi:10.1371/journal.pone.0285606)
Supplement: S3 Table — (DOCX) [file pone.0285606.s005.docx]

|  | **ID, No Supplements (n=132,481)** | **ID, Injections/tablets (n=51,690)** | **ID, Injections (n=12,720)** | ***P*-Value** |
| --- | --- | --- | --- | --- |
| **All** | 93.6 (86.4–96.9) | 90.7 (80.2–95.6) | 87.2 (72.1–94.1) | 0.34 |
| **Sex** | | | | |
| Male | 91.9 (82.6–96.2)  [n=24,753] | 83.0 (61.9–92.4)  [n=9804] | 79.5 (49.7–91.6)  [n=2797] | 0.37 |
| Female | 94.0 (87.4–97.2)  [n=107,728] | 92.3 (83.5–96.4)  [n=41,886] | 89.6 (77.0–95.3)  [n=9923] | 0.43 |
| **Age**, y | | | | |
| 16–44 | 97.0 (93.6–98.6)  [n=67,376] | 93.6 (86.3–97.0) [n=23,744] | 90.1 (77.0–95.7)  [n=4410] | 0.20 |
| 45–64 | 92.6 (84.3–96.5)  [n=40,579] | 92.2 (83.1–96.4)  [n=14,318] | 91.1 (79.2–96.2)  [n=4273] | 0.79 |
| ≥65 | 80.2 (56.9–90.9)  [n=24,526] | 83.0 (62.5–92.3)  [n=13,628] | 77.9 (46.8–90.8)  [n=4037] | 0.89 |
